# Locus-specific analysis of Transposable Elements during the progression of ALS in the SOD1^G93A^ mouse model

**DOI:** 10.1371/journal.pone.0258291

**Published:** 2021-10-06

**Authors:** Braulio Valdebenito-Maturana, Esteban Arancibia, Gonzalo Riadi, Juan Carlos Tapia, Mónica Carrasco

**Affiliations:** 1 Núcleo Científico Multidisciplinario, School of Medicine, Universidad de Talca, Talca, Chile; 2 Centre for Bioinformatics, Simulation and Modelling, CBSM, Department of Bioinformatics, Faculty of Engineering, University of Talca, Talca, Chile; 3 ANID – Millennium Science Initiative Program Millennium Nucleus of Ion Channels-Associated Diseases (MiNICAD), Centre for Bioinformatics, Simulation and Modelling, CBSM, Department of Bioinformatics, Faculty of Engineering, University of Talca, Talca, Chile; 4 School of Medicine, Universidad de Talca, Talca, Chile; "INSERM", FRANCE

## Abstract

Transposable Elements (TEs) are ubiquitous genetic elements with the ability to move within a genome. TEs contribute to a large fraction of the repetitive elements of a genome, and because of their nature, they are not routinely analyzed in RNA-Seq gene expression studies. Amyotrophic Lateral Sclerosis (ALS) is a lethal neurodegenerative disease, and a well-accepted model for its study is the mouse harboring the human *SOD1*^*G93A*^ mutant. In this model, landmark stages of the disease can be recapitulated at specific time points, making possible to understand changes in gene expression across time. While there are several works reporting TE activity in ALS models, they have not explored their activity through the disease progression. Moreover, they have done it at the expense of losing their locus of expression. Depending on their genomic location, TEs can regulate genes in cis and in trans, making locus-specific analysis of TEs of importance in order to understand their role in modulating gene expression. Particularly, the locus-specific role of TEs in ALS has not been fully elucidated. In this work, we analyzed publicly available RNA-Seq datasets of the *SOD1*^*G93A*^ mouse model, to understand the locus-specific role of TEs. We show that TEs become up-regulated at the early stages of the disease, and via statistical associations, we speculate that they can regulate several genes, which in turn might be contributing to the genetic dysfunction observed in ALS.

## Introduction

Transposable Elements (TEs) are genetic elements capable of mobilization within a genome. They can be classified into retrotransposons (that involve the reverse transcription of their mRNA) and DNA transposons (which are excised from their original location and inserted elsewhere in the genome). Because of their activity, they are found repetitively and ubiquitously in almost every genome, representing almost 50% of the human, and 40% of the mouse genomes. Due to the potentially deleterious activity of transposition in gene expression, most TEs have accumulated mutations that render them inactive and/or are repressed via epigenetic mechanisms [1]. Despite this, and contrary to the initial belief that TEs were “junk DNA”, they are now recognized as regulators of gene expression. Examples of this are TE-derived sequences having Transcription Factors binding motifs, TEs that are transcriptionally activated to generate non-coding RNAs, TEs can influence alternative splicing by becoming part of genes in order to generate novel transcript isoforms or by causing exon skipping, add binding sites for non-coding RNAs, and influence chromatin remodeling [2–4]. Overall, there is evidence showing that TEs can regulate the activity of nearby genes [2], and in some instances, they may regulate the activity of genes over hundreds of kilobases away [5]. Furthermore, Fuentes el al. recently provided further evidence of the latter, showing that some TEs can act as transcriptional enhancers, modulating the activity of distant genes [6]. Thus, either TEs that are transposition-competent or those that are already fixed in a genome, have the capability to modulate gene activity [7]. For example, expression of the LINE1 TEs in healthy brain has been associated with memory formation and neuronal plasticity by contributing to genomic mosaicism in the brain [8–10]. Moreover, TEs are thought to have contributed to central nervous system development, and amongst all somatic tissues, they seem to be more expressed in neuronal cells [11], and in Amyotrophic Lateral Sclerosis, TE expression seems to occur in both cortical and spinal neurons, the cell population most affected in that disease [12].

Amyotrophic Lateral Sclerosis (ALS) is a fatal neurodegenerative disease caused by the progressive loss of upper (motor cortex) and lower (spinal cord) motor neurons (MNs) [13]. As a consequence of MNs loss, ALS causes muscle paralysis, which eventually results in respiratory failure [13]. The *SOD1* gene was the first to be associated in a causally manner to ALS. In turn, the first stablished ALS model is the *SOD1*^*G93A*^ transgenic mouse, in which the spinal cord MNs become the most affected. [14, 15]. As in humans, it is possible to define precise stages of disease progression in mouse models, according to their genetic background. In particular, the model used in this work is the mouse carrying the transgene array of ~20 copies of the human SOD1^G93A^ mutant stablished in [16]. Despite the model replicating ALS clinical progression, it is worth mentioning that the model does not replicate in a 1:1 manner the genetic background causative of the disease [15]. Nonetheless, the SOD1^G93A^ mice model is the most extensively used to study ALS, and has been used in the development of drugs to treat the pathology. Thus, for the conditions relevant for this study, the *SOD1*^*G93A*^ mouse model, the pre-symptomatic stage is defined during the first 4–8 weeks of age, where no motor deficit signs are visible. The onset stage of the disease begins at 12 weeks of age, with distinguishable phenotypic and histopathological hallmarks that impact directly on the ability to move. Afterwards, the disease worsens (symptomatic) reaching the end stage at 17 weeks of age with severe loss of MNs, resulting in hind limb paralysis [16, 17].

To understand the changes in gene expression that takes place in ALS spinal cord tissue, Phatnani et al., performed bulk RNA-Seq assays at Weeks 4, 8, 12 and 17, in *SOD1*^*G93A*^ mice, and the corresponding Wild-Type (WT) control mice [9]. Thus, covering all of the aforementioned and well-defined *SOD1*^*G93A*^ mouse disease stages. Multiple genes and signaling pathways, such as *TGF-Beta*, were found to be affected, all potentially contributing to neurodegeneration. However, the work did not examine whether TEs become transcriptionally active through the disease progression, and whether they can potentially alter genes linked to the disease, as they focused in generating a catalogue (“fingerprint”) of gene expression and pathway changes in ALS. Earlier works have shown over-expression of the Reverse Transcriptase (RT) protein of Human Endogenous Retroviruses (HERVs) in the brain of ALS patients compared to non-ALS patients [18] and that activation of the HERV-K retrotransposon leads to neurodegeneration by affecting the murine upper and lower MNs [19]. Recent works have shown in other ALS models that TEs are active in the disease, and that chromatin becomes decondensed at LINE1 TEs throughout the genome [20], however there is conflicting evidence to whether they may contribute or not to neurodegeneration [21–24]. Moreover, none of these works have proposed putative links between genes and TEs based on their genomic location. This is mainly to the lack of use of tools that analyze TE expression at the locus level. As mentioned above, TEs can influence the activity of neighboring genes, and so far, it is unclear whether they may do so during ALS. In this work, we address this problem by using the recently published tools SQuIRE [25] and TEcandidates [26] to analyze locus-specific TE expression, in order to understand how TEs are expressed throughout the disease, and to predict potential genes that might be modulated by TEs.

## Methods

RNA-Seq data previously published, and publicly available at the Gene Expression Omnibus (GEO) database was utilized (accession GSE43879) [17]. This dataset corresponds to sequencing of the whole transcriptome derived from spinal cord tissue of *SOD1*^*G93A*^ mice and the corresponding Wild-Type (WT) control mice at Weeks 4, 8, 12 and 17. The reads were paired-end, with each pair having a read length of 36 bp (Table 1).

**Table 1:** Sample description of the RNA-Seq datasets used in our work, published by Phatnani et al (2013).

SRA Accession	Gender	Genotype	Timepoint
SRR653792	Female	SOD1^G93A^	4 weeks
SRR653793	Male	SOD1^G93A^	4 weeks
SRR653794	Female	WT	4 weeks
SRR653795	Male	WT	4 weeks
SRR653796	Female	SOD1^G93A^	8 weeks
SRR653797	Male	SOD1^G93A^	8 weeks
SRR653798	Female	WT	8 weeks
SRR653799	Male	WT	8 weeks
SRR653784	Female	SOD1^G93A^	12 weeks
SRR653785	Male	SOD1^G93A^	12 weeks
SRR653786	Female	WT	12 weeks
SRR653787	Male	WT	12 weeks
SRR653788	Female	SOD1^G93A^	17 weeks
SRR653789	Male	SOD1^G93A^	17 weeks
SRR653790	Female	WT	17 weeks
SRR653791	Male	WT	17 weeks

SRA Accession, SRA accession identifier at the Sequence Read Archive; SOD1^G93A^, mouse carrying the SOD1^G93A^ mutation; WT, wild-type control mouse.

RNA-Seq quality control was done with FastQC [27]. RNA-Seq read mapping and locus-specific TE expression analysis was done using SQuIRE [25]. The *Mus musculus* mm10 genome version was used. Differential expression analysis were done using DESeq2 [28], comparing at each time point, the *SOD1*^*G93A*^ samples with their respective Wild-Type/Control samples.

To assess the ability of SQuIRE to correctly estimate expression of young TEs at the locus level, we performed an *in silico* experiment. First, to estimate the TE Divergence from consensus sequences, we used RepeatMasker, and further processed the results using *in-house* scripts. This was needed to check if there was a bias in the Differentially Expressed TEs towards younger instances (<10% divergence from consensus sequence). Statistical analysis of this bias was performed with a Test of Given Proportions, using R, setting the significance level at 5%. [29].Then, read simulation was performed using Polyester [30], with 20x coverage for each TE, varying the read length between 36 bp, 72 bp and 108 bp (representing 1, 2 and 3 times the read length of the dataset used in this work). Afterwards, we ran SQuIRE in these simulated data sets, and evaluated the improvement in its Precision and its Recall when considering only those TEs predicted by SQuIRE that were also predicted by TEcandidates.

Classification of TEs according to their genomic location and overlap with genes was assessed with BEDtools [31]. Briefly, the overlap between TEs and exons was assessed. TEs overlapping with exons were labeled as “Exonic”. TEs not overlapping exons, were then used again, and their overlap with complete genes was assessed. TEs having overlaps at this point were labeled as “Intronic”. Finally, TEs not having any overlap in this step, were labeled as “Intergenic”. Intergenic TEs were then linked to the closest gene using BEDtools. Significant gene-TE associations were obtained using TEffectR [32].

Protein-Protein interaction networks for selected genes were obtained from the STRING database [33].

All plots were generated using ggplot2 [34].

## Results

### Differential expression of TEs using SQuIRE

**Fig 1:**
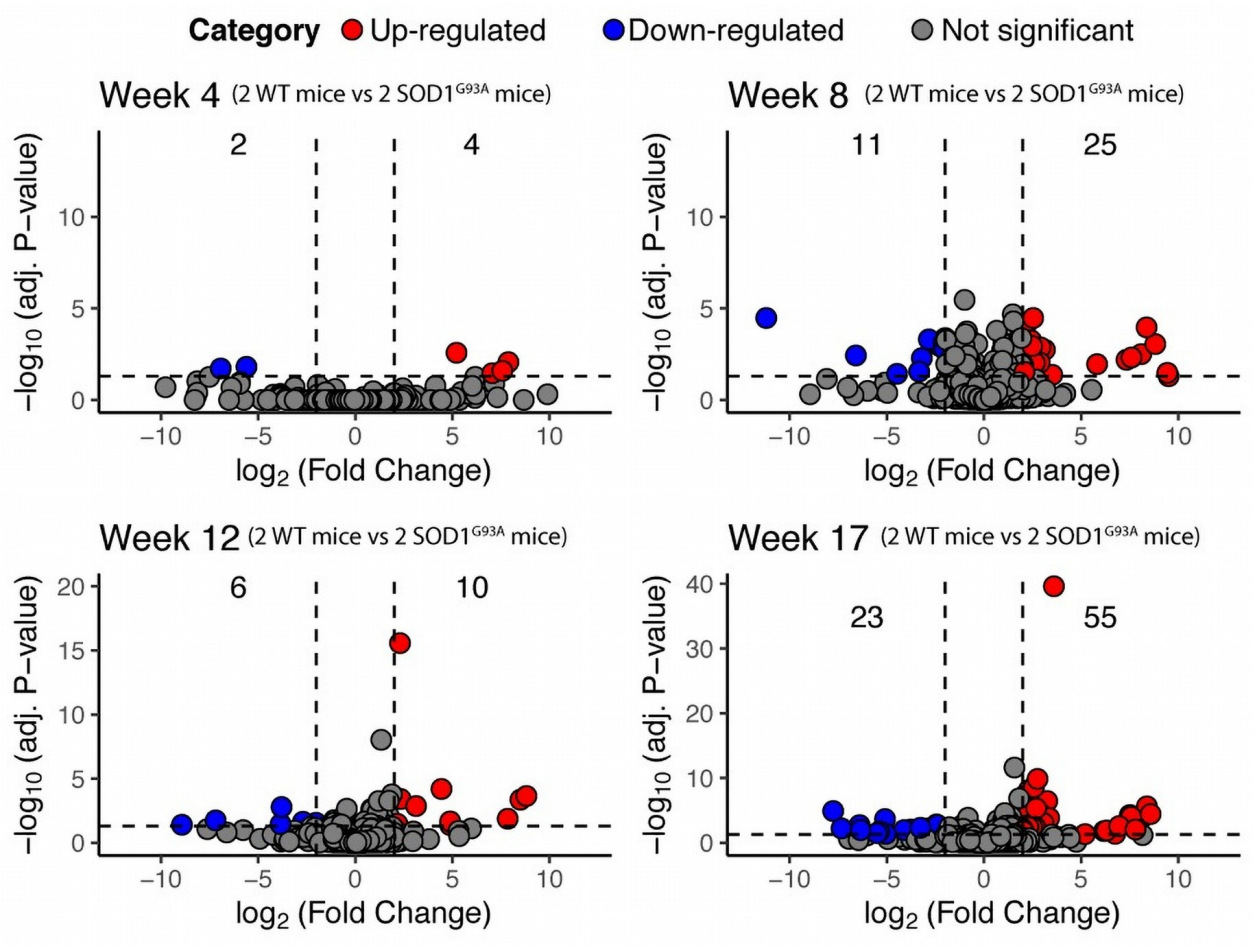
Volcano plots across the disease progression. For each plot, the log_2_(Fold Change) is shown in the x-axis, and the -log_10_(adjusted P-value) on the y-axis. The horizontal dashed line represents the adjusted P-value cutoff of 0.05, and the vertical dashed lines represent the log_2_(Fold Change) cutoffs at -2, and 2. Blue dots represent down-regulated TEs, and Red dots represent up-regulated TEs. Numbers at the upper left of each plot correspond to the number of down-regulated TEs, those at the center correspond to the TEs without significant changes in expression, and those at the upper right, to the number of up-regulated TEs. Below the number of the up-regulated and down-regulated TEs, percentages indicate the proportion of those TEs amongst all the expressed TEs found in the analyzed conditions.

After RNA-Seq quality control, the reads were aligned to the *Mus musculus* genome, in order to conduct differential expression analysis of TEs using SQuIRE and DESeq2 (Methods). To gain insights on the TEs that become up-regulated during the disease progression, the comparison between *SOD1*^*G93A*^ and WT samples was done at each time point (Fig 1).

Given that differentially expressed TEs (DE-TEs) in one time point, might not be differentially expressed in another, a time course analysis of all the DE-TEs was performed (S1 Fig). This result revealed that only a few TEs might be constantly expressed during the disease progression, with others showing a switch-like pattern of expression (i.e., up-regulated at one time point, but down-regulated at another). A caveat in this analysis is that it is unfeasible to to fully confirm this, as we have a small sample number (2 replicas per condition) and the study does not have a longitudinal study design. Overall, a longitudinal study design for this model is not possible, as animals are sacrificed at each time point. It could be argued then that the observed patterns of expression might be indicative of somatic mosaicism occurring at the different time points [35].

Previously, up-regulation of some TEs was shown in the murine SOD1^G93A^ model. This finding was limited to the L1orl elements, with others such as the L1spa not showing significant changes in expression [22]. Both of these type of L1 TEs are now classified as part of the L1Md family [36], and we were able to find 3 L1Md TEs amongst those changing their expression in the *SOD1*^*G93A*^ mice (S1 Fig). In the aforementioned work, the authors only assessed the changes in expression of 3 type of TEs via qPCR, and as they only saw the L1orl increasing its expression, they assumed that it is unlikely that TEs become up-regulated in the *SOD1*^*G93A*^ mice. In contrast to that work, due to our usage of RNA-Seq, which unlike qPCR captures expression at a genome-wide scale, we found 128 TEs with significant changes in expression, including 3 L1Md TEs, representing the same family of TEs that was previously reported to increase their expression as mentioned above. Overall, our finding confirms previously reported TEs, and adds other TEs to the repertoire of those changing their expression in the *SOD1*^*G93A*^ mice, representing further evidence towards a potential link between the mutation and TEs up-regulation.

Considering that transposition-competent TEs might generate DNA damage that might in turn be linked to neurodegeneration, we checked if there is any TEs amongst the DE-TEs that could be transpositionally active. Most of the TEs have predicted protein sequences with Stop codons, with only 36 TEs not having any Stop codon (S1 Table). Of these, 22 correspond to the SINE group, 5 to the LINE group, 6 to the LTR group and 3 to the DNA group. As SINE elements depend on the LINE elements, we inspected the latter. All LINE elements have lengths in the 43–212 bp range. The length for transposition-competent LINE is between 6,000–7,000 bp [37]. Thus, we argue that no LINE element predicted is able to transpose. A similar issue can be seen for the DNA transposons, with lengths ranging between 87–157 bp, and active elements being of around 900–1,000 bp [38], and for LTRs, with lengths ranging between 39–1,836 bp, and active elements being of around 4,000 bp [38]. Based on this, we speculate that the influence of TEs in the *SOD1*^*G93A*^ mouse model is unlikely to be related to their transposition activity. A similar finding has been previously reported by Penndorf et al., indicating that there is no significant DNA damage (indicative of transposition not occurring) in the murine *SOD1*^*G93A*^ model [22]. It is possible that across the disease progression, TEs influence gene expression via the mechanisms mentioned above, i.e., involved in non-coding RNA production, or by affecting the chromatin environment at specific regions in the genome.

### Statistical validation of expressed loci

**Fig 2:**
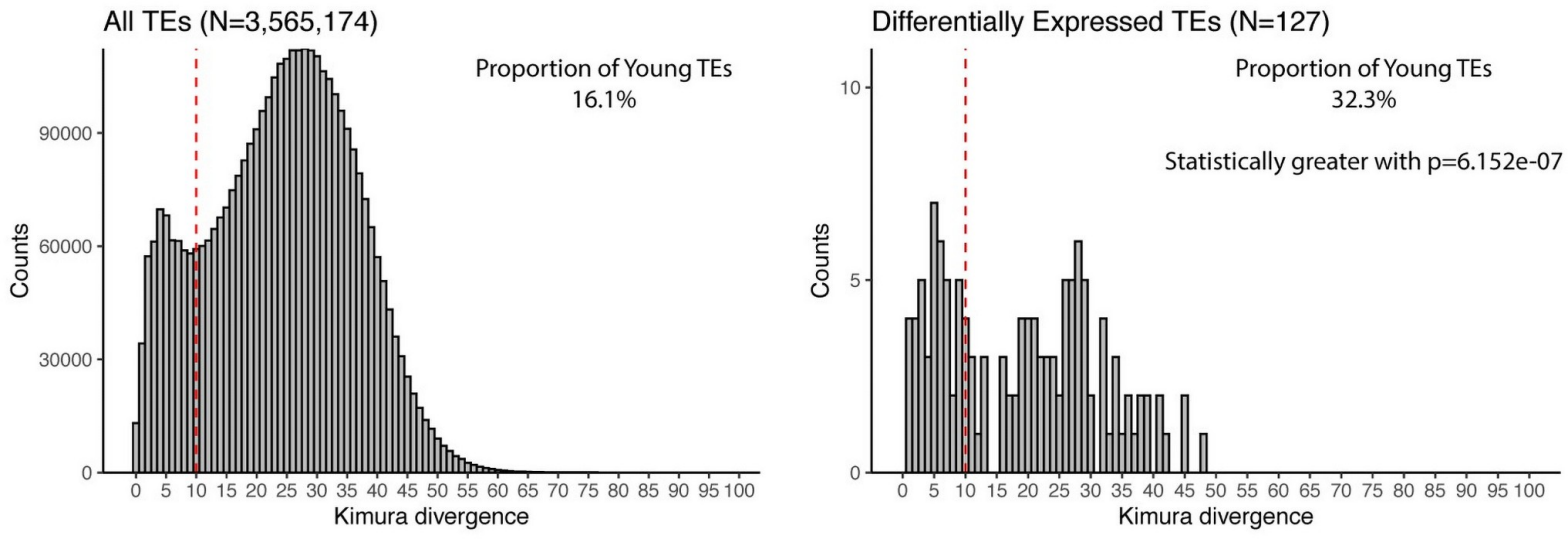
Kimura divergence histograms for *Mus musculus* TEs. “All TEs” (left) correspond to all TEs in the *Mus musculus* genome (“All TEs”, left), and DE-TEs (right) to the Differentially Expressed TEs. Dashed line at 10% correspond to the threshold to label TEs as young (≤10%) or old (>10%). The proportion of young TEs in each group is shown at the upper right corner of the respective plot.

Before stablishing associations between genes and TEs through expression, a major drawback of the RNA-Seq dataset used, is the ultra-short read length of 36 bp. While the reads are Paired-End (PE), which may improve the mappability somehow, major doubts about the certainty of the locus-specific expression of TEs can be raised. This is, particularly, an issue for younger TEs (TEs having less than 10% divergence from consensus sequences) and SQuIRE, the tool used to estimate TE expression is less accurate for these TEs [25]. To assess if there is a bias to younger TEs amongst our DE-TEs, we compared the Kimura divergence distribution of all TEs in the *Mus musculus* genome versus the same distribution of the DE-TEs (Fig 2). We found that the proportion of young TEs in the DE-TEs set was statistically greater than that of all TEs (32.3% vs 16.1%, respectively, p = 6.152e-07).

**Fig 3:**
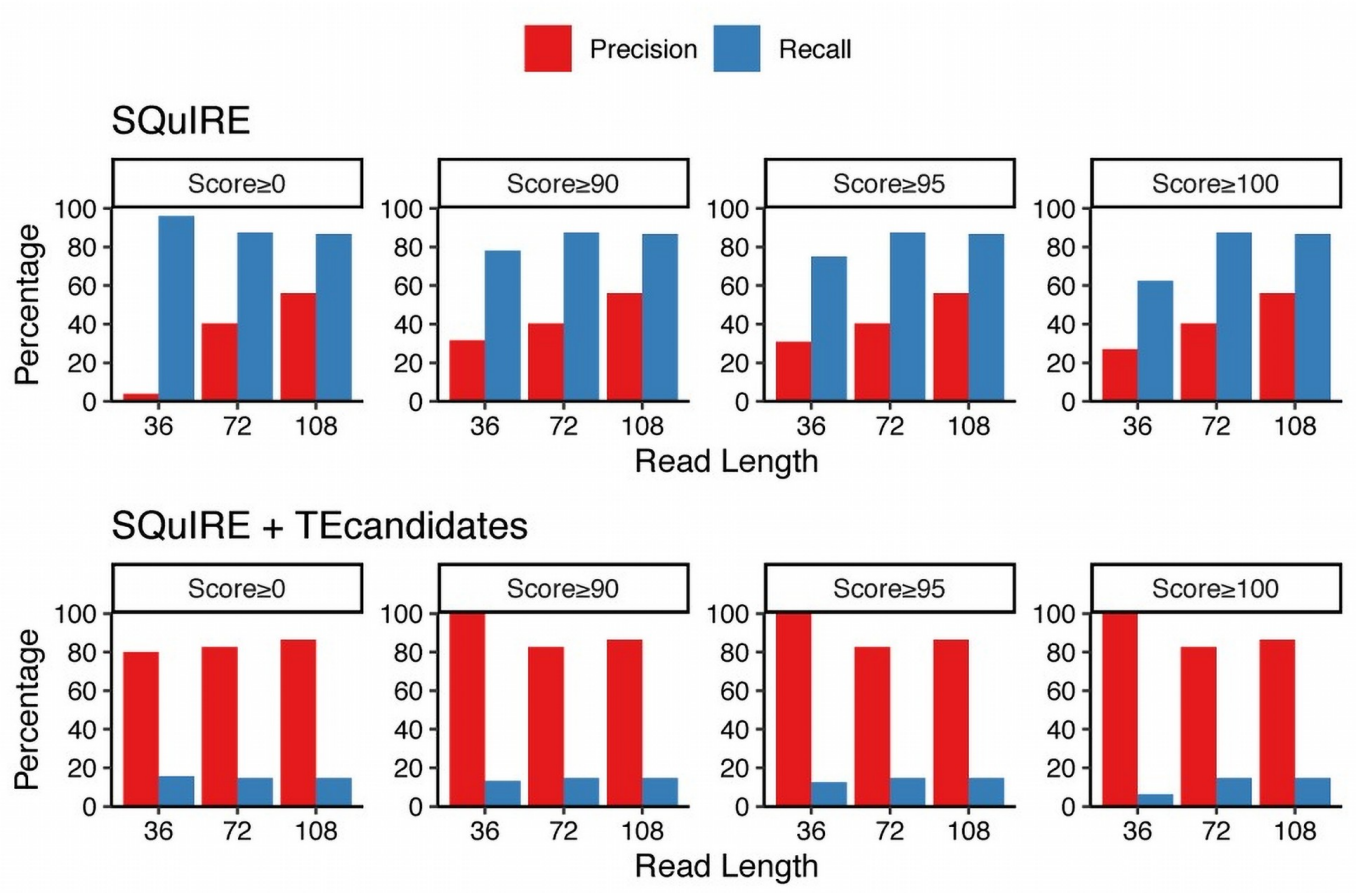
Bar plots of Precision (red) and Recall (blue) for the simulated experiments. The Upper half are the results of using SQuIRE alone, and in the lower half are the results of using SQuIRE and TEcandidates. Each bar plot corresponds to the result using a specific SQuIRE score threshold.

Unpublished results from our laboratory show that SQuIRE tends to predict a high number of results, particularly when there are young TEs. SQuIRE indirectly address this issue by reporting a score for each locus-specific TE expression. Scores equal to 100 indicate that SQuIRE is 100% confident on the expression estimate, whereas lower scores indicate the opposite, and is mainly influenced on the TEs having more multi-mapped reads aligned to them. However, we also noted that SQuIRE can estimate false positives with high scores.In order to diminish the number of false positives, we restricted the SQuIRE results using our previously published tool, TEcandidates [26]. Differently from SQuIRE, TEcandidates performs *de novo* transcriptome assembly, to generate synthetic long reads which may improve the mappability. Thus, TEcandidates is designed to predict which TE locus might be the origin of expression, and in turn, generates a lower amount of predictions. Also, TEcandidates does not estimate TE expression levels. We hypothesized that a combination of both tools might allow us to find out a more reliable subset of expressed TEs. To this end, we performed a simple experiment: we simulated reads from the DE-TEs, then we used SQuIRE to estimate TE expression with different score thresholds, and we evaluated the *Precision* (percentage of correctly predicted TEs relative to all the predicted TEs) and *Recall* (the proportion of the TEs that are correctly predicted relative to all the TEs that are indeed expressed) using SQuIRE alone and SQuIRE+TEcandidates. In this experiment we also varied the read length to see how our results would improve if we had access to a set with longer read length (Fig 3 and S2 Table).

While increments for SQuIRE’s Precision can be seen for longer read lengths, it seems that the main factor when using SQuIRE alone would be the use of its Score Threshold. Nonetheless, even for reads of length 108 nt, using the maximum Score Threshold yields a Precision of 56.1% and Recall of 86.7%. Moreover, this result suggests that for our analysis, even when using the same Score Threshold = 100, we can expect to have a Precision of only 27.1% and a Recall of 62.5%. When using the SQuIRE+TEcandidates approach, a considerable improvement in the Precision can be seen across all read lengths and across all Score Thresholds in the simulated experiments. The main drawback to this approach is that the Recall diminishes in a large amount. It is worth noting that at Score Threshold = 100, the Precision of SQuIRE+TEcandidates is greater at read length = 36, than at read lengths = 72 and 108. This is mainly due to the fact that these metrics are relative. When looking at absolute numbers, at read length = 36, 8 TEs are predicted, and 8 are correctly predicted, whereas for read length = 72, 23 are predicted, and 19 are correctly predicted (S2 Table). Taken together, these results show that using a combination of both tools might allow us to access to a subset of high-confident expressed TEs at the locus level. When applied to our results, this protocol indicates that we can be confident of the locus-specific expression of 3 TEs (S1 Fig, highlighted by arrows).

### Association of TEs with genes

**Fig 4:**
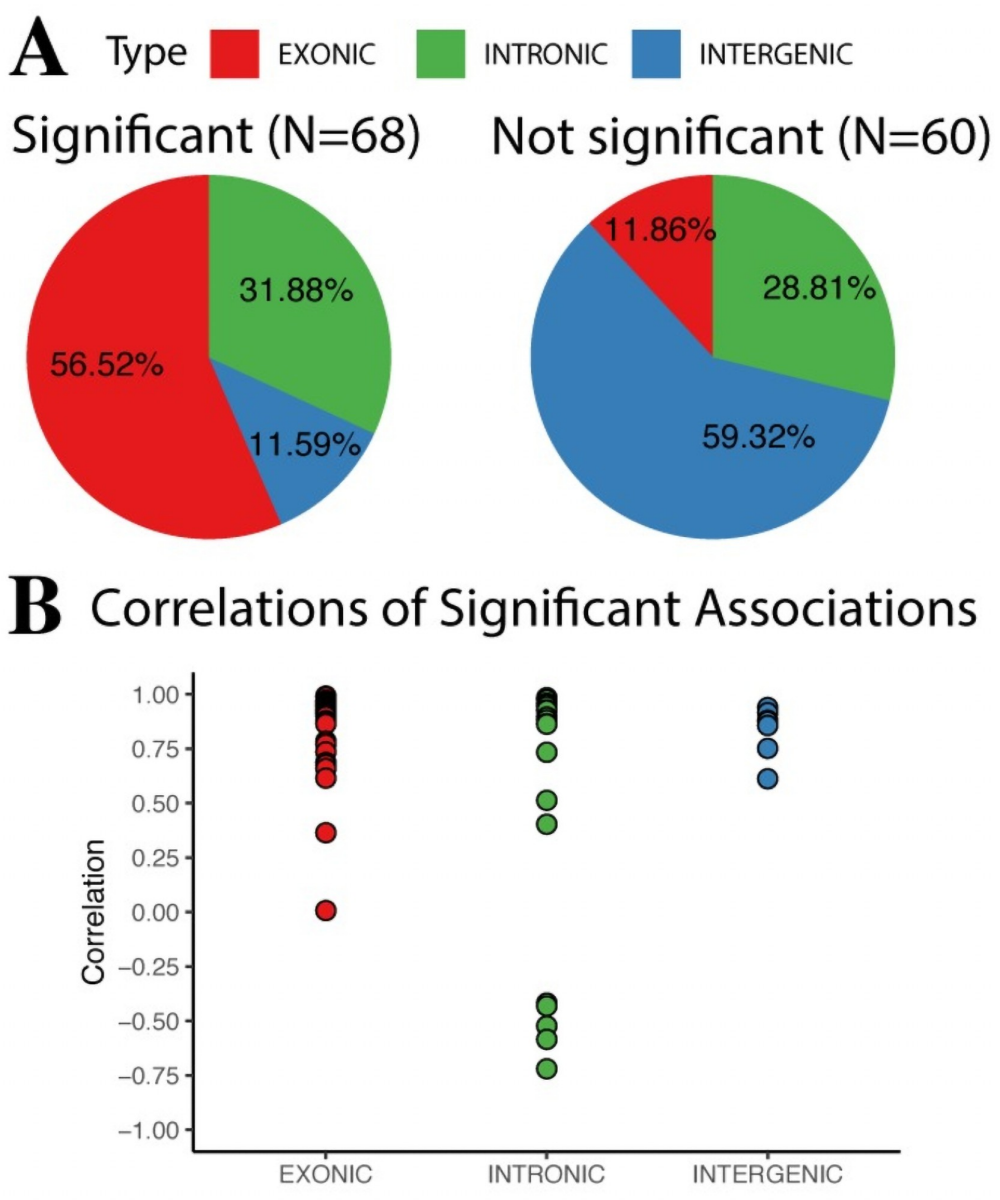
Gene-TE associations. (A) Pie charts depicting the distribution of gene-TE associations by locus type. (B) Dot plot showing the distribution of the Correlation coefficients of the statistically significant gene-TE associations by locus type.

In order to associate TEs with genes, first we divided the TEs in 3 groups according to their genomic location: Exonic, TE overlapping with a gene exon; Intronic, TE overlapping with a gene, but not its exons, and; Intergenic, TE not overlapping with any gene. This step resulted in gene-TE pairs for both Exonic and Intronic TEs. To have a gene-TE pair for Intergenic TEs, we linked them to the closest gene. Afterwards, we used TEffectR [32], which models gene expression as a response variable in function of TE expression, via linear modelling. This way, for each gene-TE pair we could statistically assess if their association is significant (S3 Table). Then, for statistically significant associations, we estimated the Pearson correlation coefficient, to measure whether a TE is positively or negatively correlated with a gene (Fig 4 and S4 Table).

About half of the DE-TEs could be significantly linked to genes, with a large proportion of these associations being of Exonic TEs. Interestingly, the opposite is seen in the not significant associations: the largest proportion corresponds to Intergenic TEs. This makes sense, considering that TEs might have effects in *cis* (in neighboring genes) and in *trans* (in genes up to thousands of nucleotides away). Thus, at least for Intergenic TEs, the approach we used might not reveal all of their regulatory potential. Nonetheless, without further data for this model this cannot be addressed. Amongst the Significant Associations, all of the Exonic TEs have a positive correlation with their corresponding Gene. This result was expected, as Exonic TEs are already genetically fixed. Thus, if its host gene is transcribed, then the TE will also appear as transcribed. For Intronic TEs, there are both positive and negative correlations. In this case, positive correlations might be indicative of co-transcription and/or intron retention, without apparently negative consequences on the host genes. On the other hand, negative correlations might be indicative of transcriptional interference. That is, the disruption of appropriate transcription of the Gene by the TE. Finally, for Intergenic TEs, all correlations were positive. Except for 1 Intergenic TE, all the others where in the vicinity of 10 kbp or less of their closest gene (S3 Table). Thus, this result suggests that Intergenic TEs might be responsible in part for the inadequate up-regulation of certain genes. Interestingly, amongst those genes we found Eny2, which is a transcription factor, whose misregulation has been speculated to be linked to cancer and neurodegenerative diseases [39].

While many of the significant gene-TE associations might indicate putative regulation mechanisms contributing to disease in the *SOD1*^*G93A*^ mice, for many of them we cannot be sure due to the complications in locus-specific TE expression estimation. Of the 3 high-confident TEs we found, only 1 of those was significantly associated to a gene. This TE, chr4|134162050|134163888|MuLV-int:ERV1:LTR|68|+, is found in an intron of the *Cep85* gene, and is negatively correlated with it. For this particular gene-TE pair we can then speculate that the TE could be disrupting the normal expression of *Cep85*.

**Fig 5:**
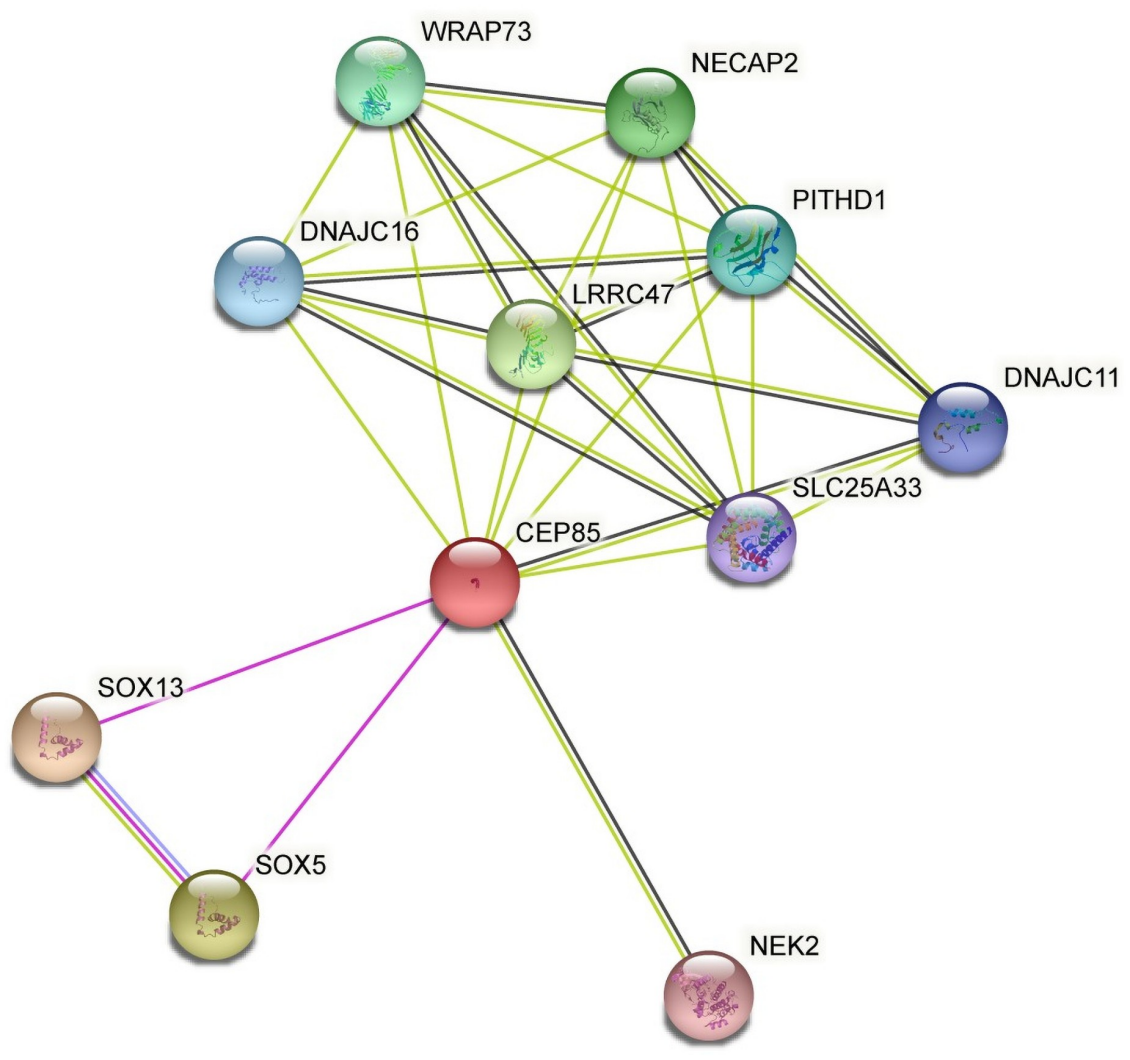
Protein-protein interaction network of *Cep85*. Green lines correspond to predicted associations, blue lines to associations reported in other databases, pink lines to experimentally confirmed associations, and black lines to co-expression links. Nodes are colored according to whether they correspond to direct associations (any color), or indirect associations (un-colored, no such association in this case).

*Cep85* (Centrosomal protein of 85 kDa) doesn’t seem to have a clear pathway associated (S4 Table). Thus, to understand the putative implications its misregulation could have, we obtained the protein-protein interactions from the STRING database (Fig 5). Then, a literature search was done to find out whether Cep85 or any of its interaction partners could be linked to neurodegeneration.

Of the interaction partners of *Cep85*, interesting evidence related to our work was found for *Nek2* (NIMA related kinase 2), *Lrrc47* (leucine rich repeat containing 47), *Slc25a33* (solute carrier family 25 member 33) and both *Sox5* and *Sox13*, which correspond to the SRY-Box Transcription Factor family. *Cep85* colocalizes with isoform A of *Nek2* (*Nek2A*) at centrosomes and forms a granule meshwork enveloping the proximal ends of centrioles. *Nek2*, in turn, has been implicated in centrosome disjunction at the onset of mitosis to promote bipolar spindle formation [40]. As the centrosome is an organelle that acts as the main organizing center for microtubules to promote bipolar spindle formation and mitotic progression, we argue that a dysregulation in the expression of *Cep85* would mean a destabilization of the cell and its death. It is worth noting that ALS MNs are post-mitotic, suggesting that there might be either an alternative role for *Cep85* in the disease, or perhaps its deregulation occurs in glial cells. *Lrrc47*, has been linked to ALS, and it has been speculated that it may play a role in protein-protein interactions in the disease [41]. It is worth noting that this is also shown in the protein-protein interacting network (Fig 5). *Lrrc47* plays a role in apoptosis, which is a well-known “pathogenic pathway” in ALS. [41]. Thus, this would also suggest that possible defects in the expression of *Cep85* might have influence on apoptosis, and in turn, contributing to the disease. *Slc25a33*, is part of the family of solute carriers, and adequate expression of this family is required for correct neuronal activity. This is mainly due to these solute carriers being responsible for neurotransmitter transport. While *Slc25a33* has not been directly linked to ALS, dysfunction in other members of the solute carrier family have [42]. It could be then suggested that in this model, impairment of *Slc25a33* might also be contributing to the disease progression. Finally, *Sox5* has been associated previously with ALS [43, 44]. While we did not find statistically significant changes of either *Sox5* or *Sox13* at the gene level, it is possible that if *Cep85* is impacting them, it is doing so at the protein level.

## Conclusions

The locus-specific role of TEs in ALS has not been explored so far, despite several works suggesting that TEs might contribute to disease. Here, by utilizing state of the art approaches for assessing the locus-specific expression of TEs, we found several putative TE-Gene regulation events. Interestingly, after all our statistical analysis, we only found 1 gene. This gene, *Cep85*, while not directly linked to the disease before, is part of an important protein-protein interaction network. Thus, the potential misregulation of this gene by a TE might indicate that TEs could play a role in the disease. As ALS MNs are post-mitotic, and *Cep85* is involved in mitotic progression, the separation of neuron populations from glia population is required to test if there is an alternative role in neurons or if *Cep85* is preferentially expressed in glial cells. Thus, future works using single cell resolution are required to better understand the potential role of *Cep85*, and to identify if there is specificity of TE expression across cell types.

The main limitation of this work is that due to the high stringency used in all of our analyses, we discarded results that could represent other gene-TE regulation events, in order to eliminate all False Positives. Based on our results, we suggest that with the advent of newer sequencing techniques that generate long reads, the locus-specific role of TEs during ALS could be more clearly elucidated in future works. Until then, our work highlights the importance of these type of analysis in expanding our knowledge in how TEs might alter gene regulation during disease.

## Supporting information

S1 Fig Transposable Elements log_2_(normalized counts) across all the mutant samples. (PDF)

S2 Fig Differentially Expressed (DE) TEs time course information. A. DE TEs locus distribution. B. DE TEs class distribution.
(PDF)

S3 Fig Heatmap of log_2_(fold changes) of DE TEs across all time points. (PDF)

S1 Table Six frame translation of differentially expressed Transposable Elements. (XLSX)

S2 Table Read simulation to address the combination of tools and parameters to keep only more confident TEs. (XLSX)

S3 Table TE-gene linear modeling. (XLSX)

S4 Table Gene TE correlations, with gene enrichment analysis. (XLSX)
