# Supplementary figures and images for: Locus-specific analysis of Transposable Elements during the progression of ALS in the SOD1G93A mouse model
Source: PLoS One. 2021 Oct 6;16(10):e0258291. doi: 10.1371/journal.pone.0258291 (PMC8494334; doi:10.1371/journal.pone.0258291)

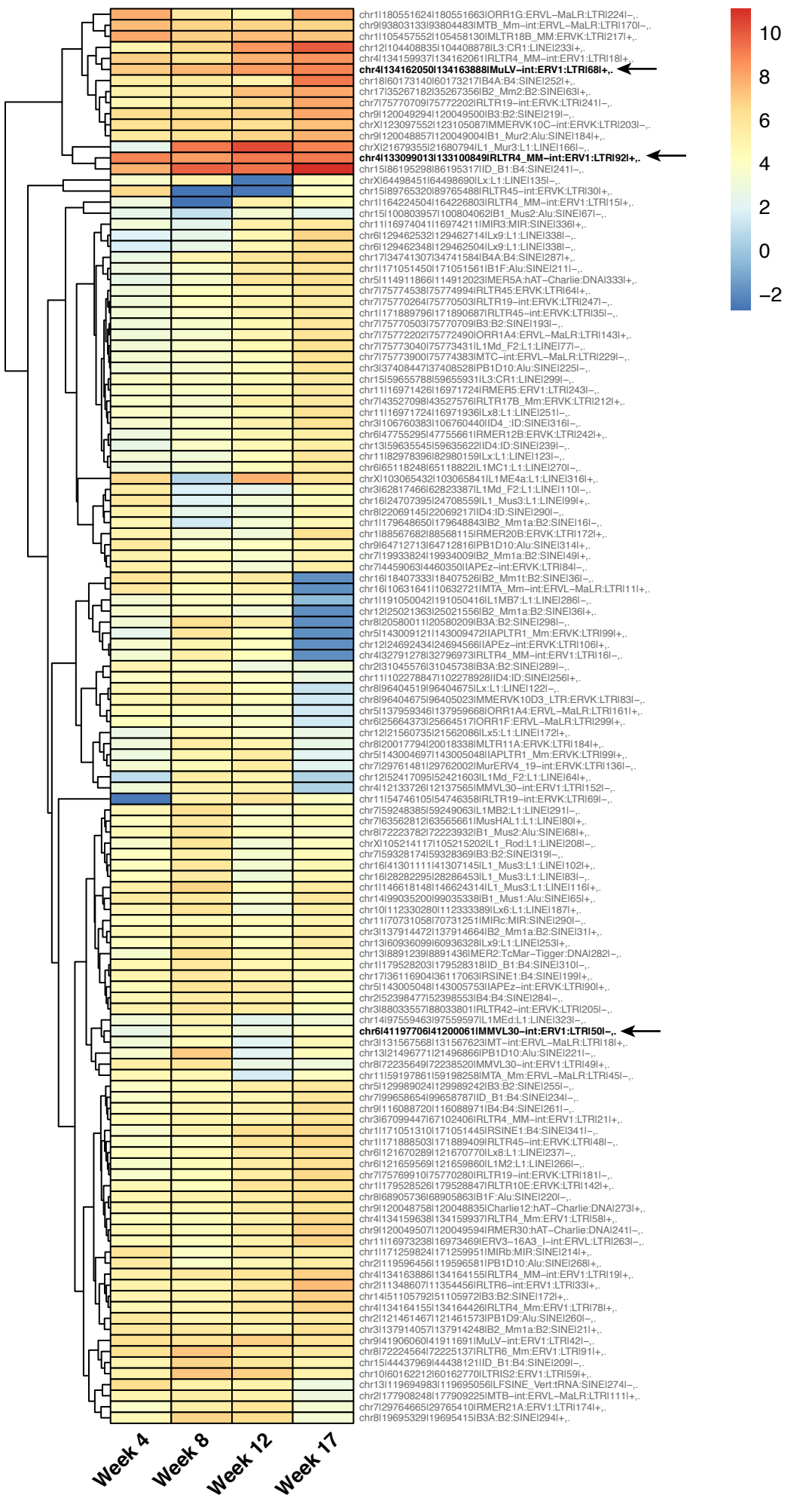

Supplement: S1 Fig — (PDF) [file pone.0258291.s001.pdf]

## A DE TEs locus distribution

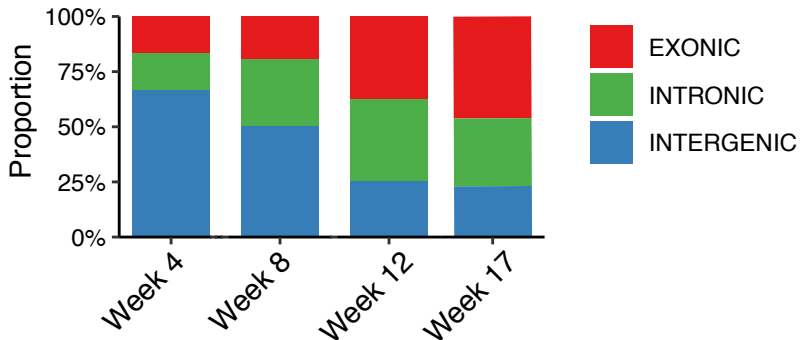

## B DE TEs class distribution

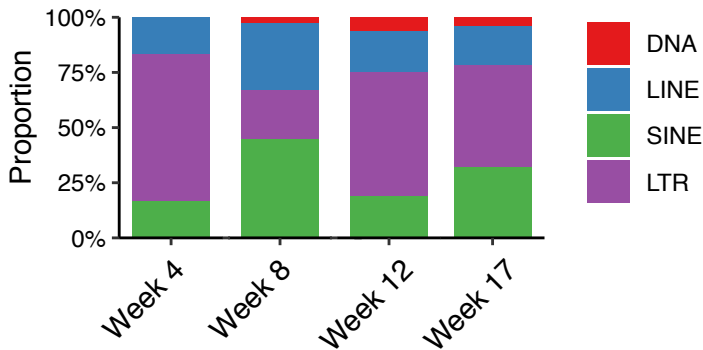

Supplement: S2 Fig — A. DE TEs locus distribution. B. DE TEs class distribution. (PDF) [file pone.0258291.s002.pdf]

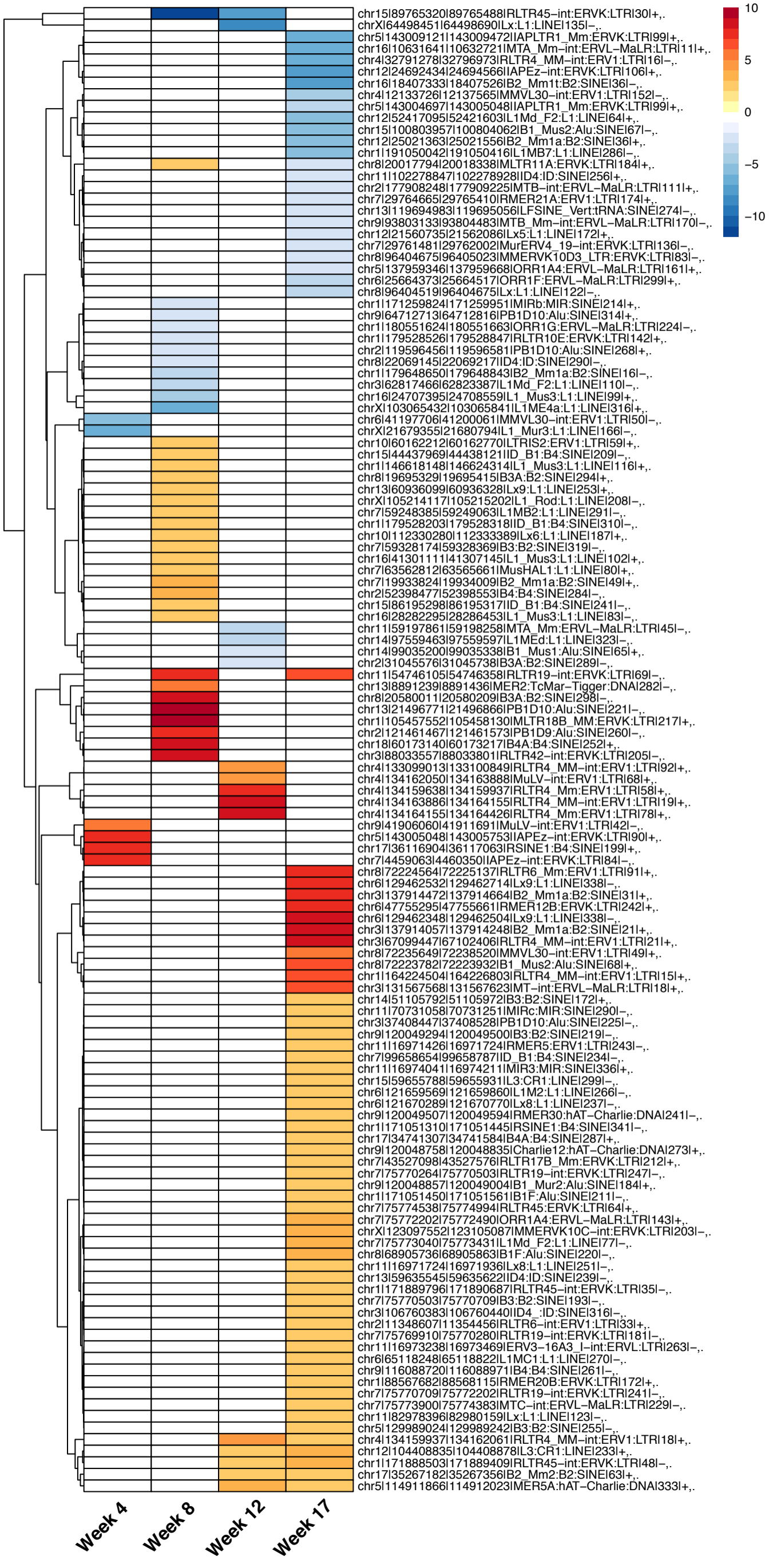

Supplement: S3 Fig — (PDF) [file pone.0258291.s003.pdf]
